# Expressions of SH3BP5, LMO3, and SNAP25 in diffuse large B‐cell lymphoma cells and their association with clinical features

**DOI:** 10.1002/cam4.753

**Published:** 2016-05-17

**Authors:** Kyoko Kobayashi, Motoko Yamaguchi, Kana Miyazaki, Hiroshi Imai, Kaori Yokoe, Ryoichi Ono, Tetsuya Nosaka, Naoyuki Katayama

**Affiliations:** ^1^ Department of Hematology and OncologyMie University Graduate School of MedicineTsuJapan; ^2^ Pathology DivisionMie University HospitalTsuJapan; ^3^ Mie University School of MedicineTsuJapan; ^4^ Department of Microbiology and Molecular GeneticsMie University Graduate School of MedicineTsuJapan

**Keywords:** CD5, diffuse large B‐cell lymphoma, LMO3, SH3BP5, SNAP25

## Abstract

Diffuse large B‐cell lymphoma (DLBCL) is clinicopathologically and genetically heterogeneous with variable clinical outcomes. We previously identified signature genes overexpressed in CD5‐positive (CD5^+^) DLBCL, which is a poor prognostic subgroup of DLBCL. To elucidate the clinical significance of the protein expression of the signature genes overexpressed in CD5^+^ DLBCL with regard to all DLBCL, not otherwise specified (NOS), 10 genes (*SH3BP5*,*LMO3*,*SNAP25*,*SYT5*,*SV2C*,*CABP1*,*FGF1*,*FGFR2*,*NEUROD1*, and *SYN2*) were selected and examined immunohistochemically with samples from 28 patients with DLBCL, NOS. Only three protein expressions, SH3BP5, LMO3, and SNAP25, were detected in DLBCL cells and then analyzed further with samples from 187 patients with DLBCL, NOS. The SH3BP5, LMO3, and SNAP25 proteins were expressed in 60% (103/173), 34% (59/175), and 46% (77/168) of DLBCL patients, respectively. These protein expressions were associated with CD5 expression, and only SH3BP5 was frequently expressed in activated B‐cell‐like DLBCL (*P *=* *0.046). Compared to the SH3BP5‐negative group, the SH3BP5^+^ group was correlated with elderly onset (>60 years, *P *=* *0.0096) and advanced‐stage disease (stage III/IV,* P *=* *0.037). The LMO3^+^ group showed a worse performance status (>1, *P *=* *0.0004). The SH3BP5^+^ group and the LMO3^+^ group had significantly worse overall survival than the negative groups (*P *=* *0.030, 0.034; respectively) for the entire group. In a subgroup analysis of patients treated with rituximab‐containing chemotherapy, there was no significant difference between groups. To the best of our knowledge, this is the first report showing the protein expressions of SH3BP5, LMO3, and SNAP25 in DLBCL cells and their clinical significance in patients with DLBCL. The SH3BP5 and LMO3 protein expressions are associated with the baseline clinical characteristics of DLBCL.

## Introduction

Diffuse large B‐cell lymphoma (DLBCL) is the largest category of aggressive lymphomas and is regarded as a clinicopathologically and genetically heterogeneous group of lymphomas 1, 2. CD5‐positive (CD5^+^) DLBCL, activated B‐cell‐like (ABC) DLBCL, and nongerminal center B‐cell‐like (non‐GCB) DLBCL are included in the 2008 WHO classification as poor prognostic subgroups of DLBCL, not otherwise specified (NOS) 2. As the variable clinical features in DLBCL result from its heterogeneity, more detailed identification of the subgroups and factors that predict its aggressiveness are urgently needed. In an effort to elucidate better therapeutic strategies, the gene mutations and aberrant protein expressions that reflect the aggressiveness of each subtype are currently being investigated 3, 4, 5.

We previously analyzed 90 patients with DLBCL, NOS by gene expression profiling (GEP) and identified the signature genes that could divide DLBCLs into two groups: a CD5^+^ group and a CD5‐negative (CD5^−^) group 6. The gene that showed most significant overexpression in CD5^+^ DLBCL was *SH3BP5* (*SH3‐domain binding protein 5*) 6. SH3BP5 was originally identified as a protein interacting with Bruton's tyrosine kinase (BTK), an essential kinase for B cell differentiation and proliferation. *SH3BP5* is also known as a signature gene of ABC DLBCL 3, 4, 5, 7; however, its protein expression in DLBCL cells and its clinical significance in DLBCL patients is unknown. Interestingly, in our previous analysis, all cases of CD5^+^ DLBCL were classified as ABC DLBCLs and the CD5^+^ ABC DLBCL signature gene set that we identified contained many neurological component‐ and function‐related genes 6, which included *LMO3* and *SNAP25*. To the best of our knowledge, there are few reports addressing neuronal genes in hematopoietic malignancies. Considering the fact that CD5^+^ DLBCL shows many aggressive clinical features 8, frequent central nervous system relapse 9, and poor prognosis 9, we speculated that some of these proteins might be useful as biomarkers for more detailed identification of the aggressive subgroups for DLBCL including CD5^+^ DLBCL. Thus, to clarify their clinical significance, we performed an immunohistochemical analysis of the relationship between their expressions in DLBCL cells and patients’ clinical features.

## Materials and Methods

### Patients

The study comprised 187 patients who were diagnosed as having DLBCL, NOS according to the 2008 WHO classification 2 between August 1993 and February 2010 at Mie University Hospital and were consecutively examined for CD5 expression by means of immunochemistry (Fig. S1). Thirty‐two out of 187 (17%) patients were CD5^+^ DLBCL. All patients had no past history of any other lymphoproliferative disorders. This study included 77 patients who were analyzed by GEP in our previous study 6. All specimens were obtained at the initial presentation, after the patients had provided informed consent.

Clinical information was obtained from the Mie University Hospital records or supplied by physicians at affiliated hospitals. All the patients were treated with similar procedures at Mie University Hospital or nearby affiliated hospitals. The institutional review board of Mie University approved this study.

### Immunohistochemistry

For this study, we selected *SH3BP5* which is the most highly expressed gene of the CD5^+^ DLBCL signature gene, and nine genes that were included in the CD5^+^ ABC DLBCL signature genes that were related to neuronal system 6: *LIM domain only 3* (*LMO3*), *Synaptosomal‐associated protein*,* 25 kDa* (*SNAP25*), *Synaptotagmin V* (*SYT5*), *Synaptic vesicle glycoprotein 2C* (*SV2C*), *Calcium binding protein 1* (*CABP1*), *Fibroblast growth factor 1* (*FGF1*), *Fibroblast growth factor receptor 2* (*FGFR2*), *Neuronal differentiation 1* (*NEUROD1*), and *Synapsin II* (*SYN2*). Immunohistochemistry was performed in frozen sections, as described previously 10. Monoclonal antibodies used in this study are listed in Table S1. Positive reactions for SH3BP5 were assessed for the presence of granular cytoplasmic reactivity. For LMO3, the presence of nuclear reactivity was regarded as positive reactions. Specimens were considered positive if more than 20% of the tumor cells showed positive reactions 10.

### Statistical analysis

Correlations between the two groups were examined with Fisher's exact test or Student's *t*‐test. Survival data were analyzed with the Kaplan‐Meier method. Overall survival (OS) was defined as the time from registration until death from any cause or until the date of the last follow‐up for patients who were alive. OS for the two groups was compared by the log‐rank test. All tests were two‐sided, with a *P* value of <0.05 indicating a significant difference. All analyses were performed using IBM SPSS Statistics 22 software (IBM Japan).

## Results

### Antigen expression in DLBCL cells

**Figure 1:**
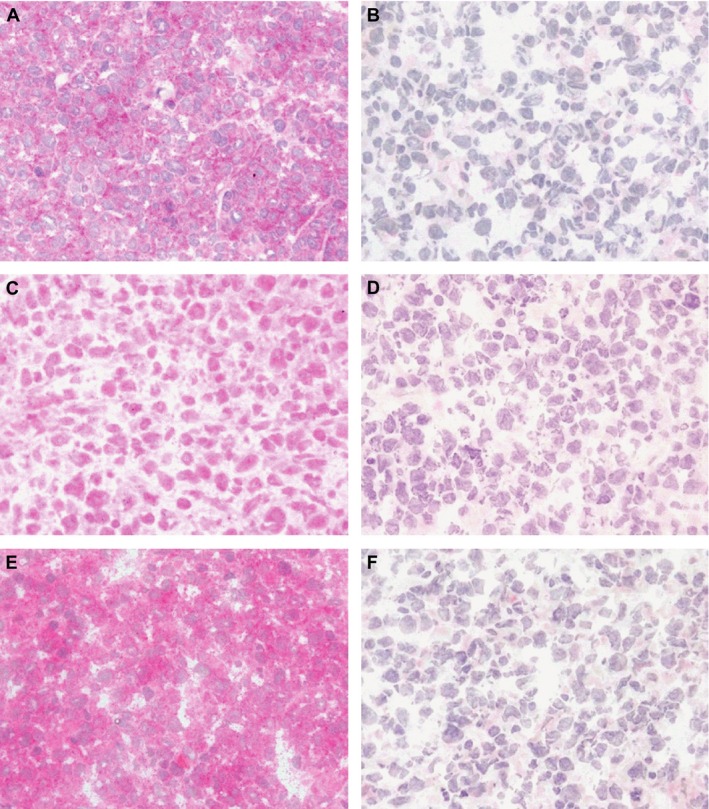
Immunohistochemistry of DLBCL tissues. (A) SH3BP5^+^ DLBCL. (B) SH3BP5^−^ DLBCL. (C) LMO3^+^ DLBCL. (D) LMO3^−^ DLBCL. (E) SNAP25^+^ DLBCL. (F) SNAP25^−^ DLBCL. SH3BP5 was positive in the cytoplasm of tumor cells showing a granular pattern. LMO3 was positive in the nuclei of DLBCL cells, and SNAP25 was positive in the cytoplasm of DLBCL cells. Original magnification was ×200 for all panels.

Among the 10 antigens, we searched for appropriate antigens which were available for identification by immunohistochemistry in frozen sections. Initially, we examined the expression of all 10 proteins in 18 patients with CD5^+^ DLBCL and in 10 patients with CD5^−^ DLBCL. The protein expressions of SH3BP5, LMO3, and SNAP25 were detected not only in CD5^+^ DLBCL cells but also in CD5^−^ DLBCL cells (Fig. 1), whereas there was no apparent expression of other seven antigens (data not shown). Thus, SH3BP5, LMO3, and SNAP25 were analyzed further.

In the whole DLBCL cohort of 187 patients, SH3BP5 was positive in 60% (103 of 173), with LMO3 positive in 34% (59 of 175) and SNAP25 positive in 46% (77 of 168). Twenty‐six patients were positive for all three antigens, and 52 patients were positive for two out of these three antigens (SH3BP5^+^ and LMO3^+^, *n* = 12; SH3BP5^+^ and SNAP25^+^, *n* = 33; LMO3^+^ and SNAP25^+^, *n* = 7).

We performed a subgroup analysis of patients with DLBCL who were included in our previous analysis of GEP 6 (Table 1). Of these patients, 23 were CD5^+^ ABC DLBCL, 42 were CD5^−^ ABC DLBCL, and 12 were CD5^−^ GCB DLBCL. CD5^+^ DLBCL patients were positive for SH3BP5, LMO3, and SNAP25 antigens more frequently than CD5^−^ DLBCL patients (87% vs. 52%, *P *=* *0.0068; 65% vs. 17%, *P *<* *0.0001; and 74% vs. 41%, *P *=* *0.018, respectively). Notably, the SH3BP5 antigen expression was more frequently detected in ABC DLBCL patients than in germinal center B‐cell‐like (GCB) DLBCL patients (70% vs. 36%, *P *=* *0.046). In a subgroup of patients with CD5^−^ ABC DLBCL, SH3BP5 was positive in 58% (19 of 33), with LMO3 positive in 11% (four of 37) and SNAP25 positive in 41% (12 of 29).

**Table 1:** Validation of SH3BP5, LMO3, and SNAP25 protein expression in DLBCL

Protein expression	CD5 expression			COO subclass		
	Positive	Negative	*P*	ABC	GCB	*P*
	*n* (%)	*n* (%)		*n* (%)	*n* (%)	
SH3BP5						
Positive	20/23 (87)	23/44 (52)	0.0068	39/56 (70)	4/11 (36)	0.046
Negative	3/23 (13)	21/44 (48)		17/56 (30)	7/11 (64)	
LMO3						
Positive	15/23 (65)	8/48 (17)	<0.0001	19/60 (32)	4/11 (36)	0.74
Negative	8/23 (35)	40/48 (83)		41/60 (68)	7/11 (64)	
SNAP25						
Positive	17/23 (74)	16/39 (41)	0.018	29/52 (56)	4/10 (40)	0.49
Negative	6/23 (26)	23/39 (59)		23/52 (44)	6/10 (60)	

COO, cell‐of‐origin; ABC, activated B‐cell‐like; GCB, germinal center B‐cell‐like.

### Clinical features according to the expression of each antigen

We analyzed 170 patients who were able to examine at least one of the three antigen expressions (SH3BP5, LMO3, and SNAP25) and had sufficient follow‐up data (Fig. S1). Clinical features at diagnosis according to the expression of each antigen are summarized in Table 2. In comparison with the SH3BP5^−^ group, the SH3BP5^+^ group was characterized by elderly onset (>60 years, 79% vs. 62%; *P *=* *0.0096) and advanced‐stage disease (stage III/IV, 46% vs. 29%; *P *=* *0.037). In the LMO3^+^ group, more patients had a poor performance status (PS) (>1, 36% vs. 11%; *P *=* *0.0004). There was no significant difference in clinical features characterized by the SNAP25 expression.

**Table 2:** Correlation between patient characteristics and SH3BP5, LMO3, and SNAP25 expression

Characteristics	SH3BP5			LMO3			SNAP25		
	+*n* = 100	−*n* = 68	*P*	+*n* = 58	−*n* = 106	*P*	+*n* = 74	−*n* = 90	*P*
	*n* (%)	*n* (%)		*n* (%)	*n* (%)		*n* (%)	*n* (%)	
Age at diagnosis, years									
Median	70	65	0.0096	71	68	0.71	68	69	0.16
Range	43–92	23–86		30–91	23–92		41–92	23–91	
≤60 years	21 (21)	27 (40)		14 (24)	30 (28)		16 (22)	29 (32)	
>60 years	79 (79)	41 (60)		44 (76)	76 (72)		58 (78)	61 (68)	
Sex									
Male	55 (55)	41 (60)	0.53	32 (55)	61 (58)	0.87	45 (61)	47 (52)	0.34
Female	45 (45)	27 (40)		26 (45)	45 (42)		29 (39)	43 (48)	
Stage									
I–II	54 (54)	48 (71)	0.037	31 (53)	65 (61)	0.41	39 (53)	58 (64)	0.15
III–IV	46 (46)	20 (29)		27 (47)	41 (39)		35 (47)	32 (36)	
PS									
0 or 1	83 (83)	53 (78)	0.43	37 (64)	94 (89)	0.0004	57 (77)	75 (83)	0.33
>1	17 (17)	15 (22)		21 (36)	12 (11)		17 (23)	15 (17)	
Extranodal sites									
0 or 1	88 (88)	60 (88)	1	49 (84)	94 (89)	0.47	66 (89)	78 (87)	0.81
>1	12 (12)	8 (12)		9 (16)	12 (11)		8 (11)	12 (13)	
Serum LDH									
Normal	46 (46)	39 (57)	0.16	23 (40)	57 (54)	0.10	33 (45)	49 (54)	0.27
Elevated	54 (54)	29 (43)		35 (60)	49 (46)		41 (55)	41 (46)	
IPI risk categories									
Low	64 (64)	48 (71)	0.41	33 (57)	74 (70)	0.12	44 (59)	64 (71)	0.14
High	36 (36)	20 (29)		25 (43)	32 (30)		30 (41)	26 (29)	

PS, performance status; LDH, lactate dehydrogenase; IPI, international prognostic index.

### Survival analysis

Clinical information prior to first‐line treatment and follow‐up was available for 168 patients. Treatment consisted of chemotherapeutic regimens including anthracycline for 152 patients and without anthracycline for three patients. The most popular chemotherapeutic regimen was cyclophosphamide, doxorubicin, vincristine, and prednisolone (CHOP), which was selected as the initial treatment for 124 patients. Nineteen patients were treated by pirarubicin, cyclophosphamide, vincristine, and prednisolone (THP‐COP) as the initial treatment. Forty‐five patients received chemotherapy with rituximab in the first‐line therapy, and 10 patients with localized disease were treated with radiotherapy or surgical resection alone as the first‐line therapy. Three patients did not receive any treatment because of their poor PS, and all died of primary disease.

**Figure 2:**
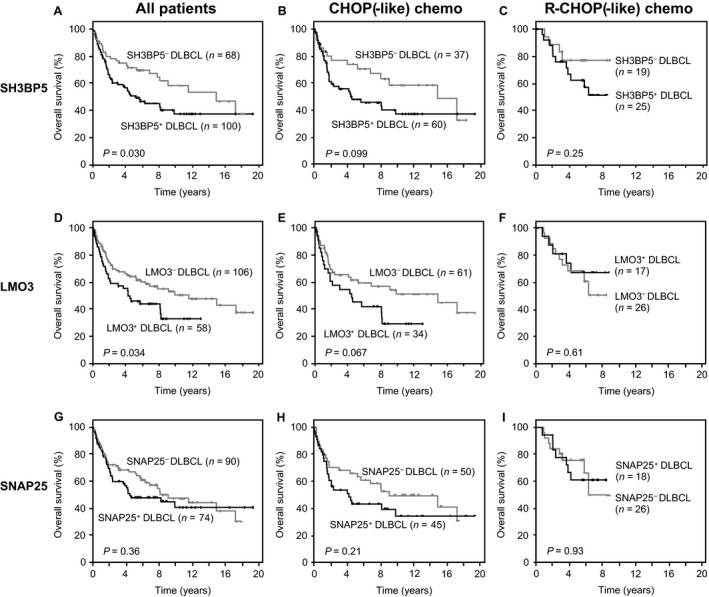
OS for patients with DLBCL. (A) All patients were analyzed for SH3BP5 expression. (B) Patients treated with CHOP(‐like) chemotherapy were analyzed for SH3BP5 expression. (C) Patients treated with R‐CHOP(‐like) chemotherapy were analyzed for SH3BP5 expression. (D) All patients were analyzed for LMO3 expression. (E) Patients treated with CHOP(‐like) chemotherapy were analyzed for LMO3 expression. (F) Patients treated with R‐CHOP(‐like) chemotherapy were analyzed for LMO3 expression. (G) Patients were analyzed for SNAP25 expression. (H) Patients treated with CHOP(‐like) chemotherapy were analyzed for SNAP25 expression. (I) Patients treated with R‐CHOP(‐like) chemotherapy were analyzed for SNAP25 expression. chemo, chemotherapy.

With a median follow‐up of 82 months in the 168 patients, the SH3BP5^+^ group had significantly worse OS than the SH3BP5^−^ group (*P *=* *0.030, 5‐year OS; 51% and 71%, respectively; Fig. 2A), and the LMO3^+^ group also had significantly worse OS than the LMO3^−^ group (*P *=* *0.034, 5‐year OS; 46% and 64%, respectively; Fig. 2D). In contrast, there was no significant difference in OS according to the SNAP25 expression (Fig. 2G).

Next, we analyzed OS in three subgroups according to the following first‐line treatment: CHOP and THP‐COP chemotherapy (CHOP[‐like] chemotherapy; *n* = 98), CHOP‐like chemotherapy with rituximab (R‐CHOP[‐like] chemotherapy; *n* = 45), and others. In the CHOP(‐like) chemotherapy group, patients with SH3BP5^+^ DLBCL showed a trend toward shorter OS than those with SH3BP5^−^ DLBCL (*P *=* *0.099, 5‐year OS; 48% [95% CI; 34–60%] and 74% [95% CI; 55–86%], respectively; Fig. 2B), and OS for the patients with LMO3^+^ DLBCL also showed a trend toward reduced OS compared to those with LMO3^−^ DLBCL (*P *=* *0.067, 5‐year OS; 46% [95% CI; 28–61%] and 61% [95% CI; 47–73%], respectively; Fig. 2E). In the R‐CHOP(‐like) chemotherapy group, there was no significant differences between the groups (Fig. 2C and F). There were no significant differences in OS according to the SNAP25 expression (Fig. 2H and I).

## Discussion

*SH3BP5*,* LMO3*, and *SNAP25* were the signature genes of CD5^+^ DLBCL in our previous study 6, and their protein expressions were significantly associated with CD5 expression. In terms of cell‐of‐origin classification, *SH3BP5* is known to be a signature gene of ABC DLBCL 3, 4, 5, 7, and we validated the SH3BP5 protein expression in ABC DLBCL. In addition, the SH3BP5 expression was observed in 52% of CD5^−^ DLBCL patients, which is a reasonable result because a portion of CD5^−^ DLBCL can be classified as ABC DLBCL. In fact, SH3BP5 was positive in 58% of CD5^−^ ABC DLBCL patients in a subgroup analysis performed in this study.

Our study revealed that the SH3BP5 and LMO3 protein expressions correlated with the baseline clinical characteristics of DLBCL. SH3BP5^+^ DLBCL correlated with elderly onset and advanced‐stage disease, and LMO3^+^ DLBCL patients showed a worse PS. Because these characteristics were included in International Prognostic Index risk factors 11, these results suggest that the SH3BP5 and LMO3 protein expressions in DLBCL correlate with the aggressiveness of DLBCL.

Patients with SH3BP5^+^ DLBCL and LMO3^+^ DLBCL who were treated with chemotherapy without rituximab showed worse OS than patients with SH3BP5^−^ DLBCL and LMO3^−^ DLBCL; however, OS for patients treated with rituximab‐containing chemotherapy was sufficiently improved regardless of the expression of these two proteins. These results suggest that the SH3BP5 and LMO3 protein expressions may be related to the molecular pathogenesis of DLBCL and that rituximab addition can overcome the negative effect of the expression of these two proteins. Similar data regarding the loss of prognostic value for DLBCL patients treated with R‐CHOP have been reported 12, 13, 14. Rituximab mediates drug‐induced apoptosis via down‐regulation of several signaling pathways and chemosensitization of non‐Hodgkin's lymphoma B‐cells 15. These effects of rituximab may also cause the difference of OS in this study as previously reported.

SH3BP5 interacts with BTK as a negative regulator 16 in normal B cells. In this regard, the function of SH3BP5 resembles that of ibrutinib, a BTK inhibitor that is highlighted for its remarkable antitumor effect in B‐cell malignancies 17, 18. SH3BP5 also interacts with c‐Jun NH2‐terminal kinase (JNK) 19, which is required for survival and proliferation of B‐cell lymphoma cells 20, 21. The endogenous level of SH3BP5 positively regulates JNK 22, 23; however, the overexpressed SH3BP5 inhibits JNK 24. If SH3BP5 in DLBCL cells acts similarly as that in normal B cells, these findings suggest that SH3BP5 overexpression in DLBCL patients might be associated with a favorable prognosis. However, our immunohistochemistry results showed that SH3BP5 expression is associated with aggressive clinical features. As for this discrepancy, we assumed the involvement of genomic mutation at the locus of *SH3BP5*; however, no potentially pathogenic mutation was found, at least, in its exon regions by sequencing of available 17 DNA samples (data not shown). Although mutation analysis covering the entire *SH3BP5* locus in other DLBCL cohorts is needed, another possibility is that the molecular mechanism of inhibition of BTK and JNK by SH3BP5 in DLBCL cells may be different from that in normal B cells. Further studies of SH3BP5 in DLBCL cells, such as functional analysis regarding interactions with BTK and JNK, will contribute to elucidating the detailed molecular mechanisms of the progression of DLBCL.

LMO3, a member of the LMO family, was originally described as expressed only in the brain and spinal cord 25, and LMO3 overexpression predicts a poor prognosis in neuroblastoma 26. Recently, aberrant *LMO3* expression in other various types of malignant cells has been reported 27, 28, 29. Our samples examined also did not include any neuronal tissue, so that the further investigation of the LMO3 expression is valuable for understanding its exact role in both normal and tumor cells. LMO2, also a member of the LMO family, is known as a biomarker of GCB DLBCL 30, 31. Several different characteristics for LMO2 and LMO3 have been described 25; however, our results of association with aggressive clinical features also suggest that LMO3 might have some relevant molecular mechanism in DLBCL cells, as LMO2 does.

For SNAP25, we could not find any correlation between clinical features and its expression. SNAP25 belongs to a family essential for synaptic and secretory vesicle exocytosis 32. To the best of our knowledge, there is no report of SNAP25 expression in malignancies. Clinical significance of SNAP25 expression in DLBCL cells remains unknown.

We performed immunohistochemistry with frozen samples because several antibodies used in this study were suitable only for immunohistochemistry with frozen sections. We confirmed that the anti‐SH3BP5 antibody used in this study is applicable for FFPE samples. Because we selected only one antibody for each protein for immunohistochemistry, further studies using other antibodies are needed to confirm our results regarding the seven proteins.

In conclusion, to the best of our knowledge, this is the first report showing SH3BP5, LMO3, and SNAP25 protein expressions in DLBCL cells and their clinical significance in patients with DLBCL, NOS. Our results suggest that further investigation especially into SH3BP5 is valuable for elucidation of more detailed molecular pathology of DLBCL. Further studies of LMO3 are warranted for a better understanding of its exact role in tumor cell differentiation and proliferation.

## Conflict of Interest

None declared.

## Supporting information


**Table S1.** Ten genes and corresponding antibodies used in the study.

**Figure S1.** Flowchart of patient selection.
