# Supplementary material for: Expressions of SH3BP5, LMO3, and SNAP25 in diffuse large B‐cell lymphoma cells and their association with clinical features
Source: Cancer Med. 2016 May 17;5(8):1802–9. doi: 10.1002/cam4.753 (PMC4873606; doi:10.1002/cam4.753)
Supplement: Supplementary file 1 — Table S1. Ten genes and corresponding antibodies used in the study. [file CAM4-5-1802-s002.docx]

**Table S1.** 10 genes and corresponding antibodies used in the study.

| Genes | Antibodies | Resources | Dilution |
| --- | --- | --- | --- |
| *SH3BP5* | Anti-SH3BP5 antibody (ab56603) | abcam | x 1000 |
| *LMO3* | LMO3 (C-14) | Santa Cruz Biotechnology | x 50 |
| *SNAP25* | Anti-SNAP25 antibody [SP12] | abcam | x 1000 |
| *SYT5* | Synaptotagmin V (N-17) | Santa Cruz Biotechnology | x 50 |
| *SV2C* | SV2C (P-20) | Santa Cruz Biotechnology | x 50 |
| *CABP1* | CABP1 Antibody (C-term) | ABGENT | x 20 |
| *FGF1* | Anti-FGF1 antibody (ab54677) | abcam | x 160 |
| *FGFR2* | Human FGF R2 Antibody (MAB6843) | R&D SYSTEMS | x 20 |
| *NEUROD1* | Neuro D (A-10) | Santa Cruz Biotechnology | x 50 |
| *SYN2* | Synapsin II a (1) | Santa Cruz Biotechnology | x 50 |
