# Supplementary figures and images for: Expressions of SH3BP5, LMO3, and SNAP25 in diffuse large B‐cell lymphoma cells and their association with clinical features
Source: Cancer Med. 2016 May 17;5(8):1802–9. doi: 10.1002/cam4.753 (PMC4873606; doi:10.1002/cam4.753)

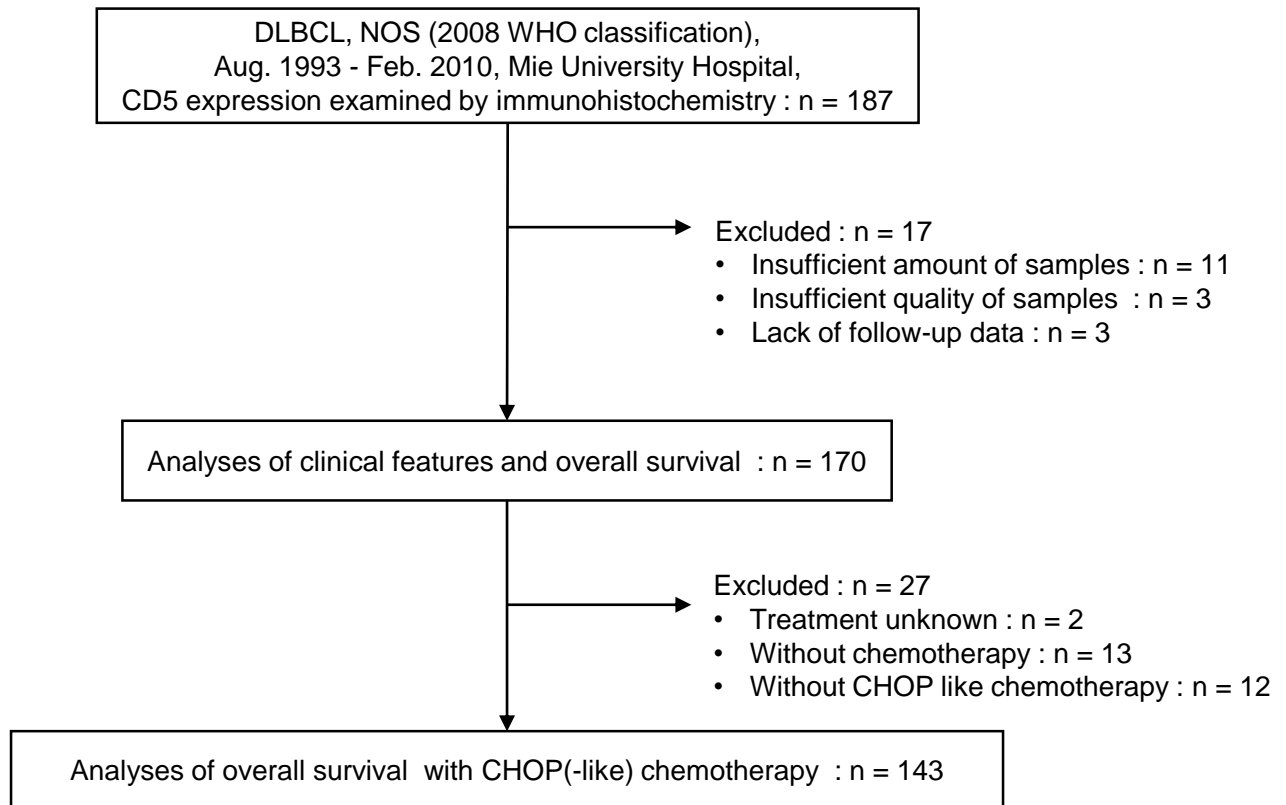

**Figure S1.** Flow chart of patient selection.

Supplement: Supplementary file 2 — Figure S1. Flowchart of patient selection. [file CAM4-5-1802-s001.pdf]
